# The Potential of CD16 on Plasma-Derived Exosomes as a Liquid Biomarker in Head and Neck Cancer

**DOI:** 10.3390/ijms21113739

**Published:** 2020-05-26

**Authors:** Linda Hofmann, Sonja Ludwig, Patrick J. Schuler, Thomas K. Hoffmann, Cornelia Brunner, Marie-Nicole Theodoraki

**Affiliations:** 1 Department of Otorhinolaryngology, Head and Neck Surgery, Ulm University Medical Center, 89075 Ulm, Germany; linda.hofmann@uni-ulm.de (L.H.); patrick.schuler@uniklinik-ulm.de (P.J.S.); t.hoffmann@uniklinik-ulm.de (T.K.H.); cornelia.brunner@uniklinik-ulm.de (C.B.); 2 Department of Otorhinolaryngology, Head and Neck Surgery, University Hospital Mannheim, 68167 Mannheim, Germany; sonja.ludwig@umm.de

**Keywords:** exosomes, HNSCC, liquid biomarker, CD16

## Abstract

Head and neck squamous cell carcinomas (HNSCC) are highly immune suppressive and aggressive malignancies. As part of the tumor microenvironment, exosomes contribute to this immune suppression. The Fc receptor CD16 is widely expressed on monocytes, neutrophils, and natural killer (NK) cells and is involved in antibody-dependent cell-mediated cytotoxicity (ADCC). Here, surface levels of CD16 on total exosomes and tumor-derived exosomes (TEX) from plasma of HNSCC patients were analyzed regarding their potential as liquid biomarkers for disease stage. Exosomes were isolated from plasma using mini size exclusion chromatography. TEX were enriched by immune affinity capture with CD44v3 antibodies. On-bead flow cytometry was used to measure CD16 levels on total exosomes and TEX. The results were correlated with clinicopathological parameters. Total exosomes from HNSCC patients had significantly higher CD16 levels compared to TEX. Further, CD16 surface levels of total exosomes, but not TEX, correlated with clinicopathological parameters. Patients with advanced tumor stages T3/4 and Union for International Cancer Control (UICC) stages III/IV had significantly higher CD16 levels on total exosomes compared to patients with early tumor stages T1/2 and UICC stages I/II, respectively. Overall, CD16 positive exosomes have the potential as liquid biomarkers for HNSCC tumor stage and aggressiveness.

## 1. Introduction

Head and neck squamous cell carcinomas (HNSCC) account for the sixth most common cancer worldwide and are characterized by profound immune suppression. In HNSCC, dysregulated and functionally impaired immune cells are commonly reported, including monocytes, neutrophils, natural killer (NK), and T cells [1,2,3,4,5,6]. CD16 (FcγRIIIA) belongs to the Fcγ receptor (FcγR) family and is widely expressed on various immune cell-types [7]. In particular, CD16 enables NK cells to detect and kill antibody-coated cells by antibody-dependent cell-mediated cytotoxicity (ADCC) [8]. Several therapeutic approaches are based on monoclonal antibodies (mABs), such as cetuximab, with the aim to induce ADCC [9,10].

As part of the communication network between tumor cells and immune cells within the tumor microenvironment (TME), extracellular vesicles (EVs), especially exosomes, play a pivotal role in immune suppression and the regulation of tumor progression [11,12,13]. Exosomes are small-sized (30–150 nm) EVs that are released by all cell types and mediate intercellular communication [14]. Exosomes differ from other EVs by their origin-unique cargo, as their biogenesis process in the endosomal compartment allows them to recapitulate the molecular characteristics of the parental cell [15]. Due to this fact and their ability to circulate freely in all body fluids, the role of exosomes as non-invasive liquid biomarkers is emerging. HNSCCs are avid exosome producers and the plasma of HNSCC patients is enriched in exosomes [16]. Our group has recently shown that those exosomes have great potential as liquid biomarkers not only for disease activity and tumor stage but also for the level of immune suppression and therapy monitoring [17,18,19,20]. Capture techniques based on immunoaffinity allow for the separation of exosomes according to their cellular origin to analyze distinct exosome subsets concerning their role in cancer progression and their clinical impact [18,19]. However, the molecular heterogeneity of HNSCC makes it difficult to identify tumor markers for selective enrichment of tumor-derived exosomes (TEX). We recently evaluated CD44v3 as a tumor-associated protein to enrich TEX from plasma of HNSCC patients [21]. CD44v3 overexpression has been associated with tumor progression and metastatic potential in HNSCC [22,23,24]. In our previous study, the molecular profiles of CD44v3(+) TEX correlated with clinicopathological parameters and thus represented a potential biomarker of HNSCC activity and progression. However, not only TEX, but also exosomes derived from immune cells, are important for the modulation of the TME. As we have shown previously, T cell-derived CD3(+) exosomes carry immunoregulatory molecules which inform about the functionality of parent T cells and significantly correlate not only with clinicopathological parameters [19] but also with response to immune therapy [18].

Due to the importance of CD16 in a functional anti-tumor immune response, in this study, we investigate the relevance of CD16 on exosomes from plasma of HNSCC patients as liquid biomarkers for disease status.

## 2. Results

### 2.1. Characterization of Exosomes

**Figure 1:**
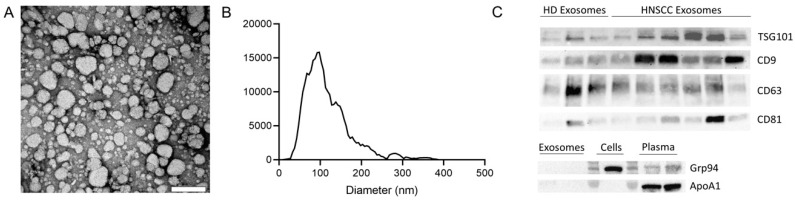
Characterization of exosomes isolated from plasma. (**A**) Representative transmission electron microscopy (TEM) image of exosomes. Scalebar = 200 nm. (**B**) Representative size distribution of exosomes measured by nanoparticle tracking analysis (NTA). (**C**) Exosomes derived from plasma of healthy donors (HD) and head and neck squamous cell carcinoma (HNSCC) patients were analyzed by Western blot for the presence of exosome specific markers using antibodies against CD63 and CD81 under non-reducing conditions and antibodies against CD9 and TSG101 under reducing conditions. Western blot analysis for the negative marker Grp94 and the apolipoprotein Apo1A was also performed for exosomes, cells, and plasma.

Exosomes isolated by mini size exclusion chromatography (SEC) were evaluated for morphology and shape by transmission electron microscopy (TEM), for size by nanoparticle tracking analysis (NTA), and for their cellular origin and purity by Western blot analysis. TEM images showed the vesicular morphology of isolated exosomes (Figure 1A) and NTA confirmed a size range of 30 to 150 nm with an average diameter of 100 nm (Figure 1B). Further, both endosomal markers (e.g., TSG101) and other vesicle-associated proteins such as CD9, CD63, and CD81 were found to be present in the exosome preparation from plasma of both healthy donors (HDs) and HNSCC patients (Figure 1C). In contrast, ApoA1 and Grp94 were absent in exosome preparations (Figure 1C). Of note, levels of exosome specific proteins showed interindividual differences. However, this interindividual variability is commonly observed in patient-derived exosomes but also between different tumor cell lines [16,25].

These methods for confirmation of the exosomes nomenclature are routinely followed as described in detail in our previous publications [16,26,27,28]. They follow the minimal information for studies of extracellular vesicles (MISEV) 2018 guidelines for the definition of EVs [29]. Using these criteria, we are confident that the EVs we isolate from plasma or cell culture supernatants are exosomes.

### 2.2. Expression Levels of CD16 in Cell Lines and Cell Line-Derived Exosomes

**Figure 2:**
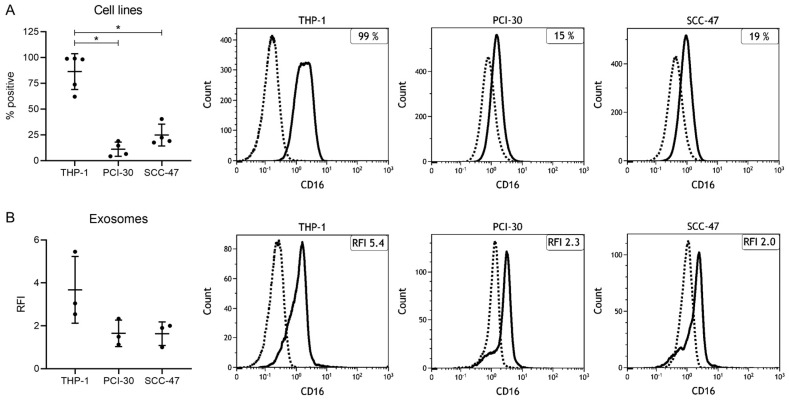
CD16 levels on cells and cell-line derived exosomes. (**A**) The monocytic cell line THP-1 and the two HNSCC cell lines PCI-30 and SCC-47 were stained for CD16. (**B**) Exosomes produced from the three cell lines were stained for CD16 and surface levels are shown as relative fluorescent intensity (RFI) compared to an appropriate isotype control. Bars represent mean with standard deviation (SD) from n = 5 (**A**, THP-1) or n = 4 (**A**, PCI-30, SCC-47) and n = 3 (**B**) independent flow cytometry experiments. *p* values were determined by Mann–Whitney test, with * corresponding to *p* ≤ 0.05. Representative flow cytometry histograms depicting CD16 levels on the individual cell lines (**A**) and their corresponding exosomes (**B**) are shown. The solid line represents the CD16 signal, the dashed line represents the unstained (**A**) or isotype (**B**) control.

The monocytic THP-1 cell line was found to be strongly positive for CD16 (86% ± 17%), whereas the HNSCC cell lines PCI-30 and SCC-47 exhibited significantly lower levels (11% ± 7% and 25% ± 11%, Figure 2A). Next, exosomes isolated from cell culture supernatants were investigated for CD16 surface levels by on-bead flow cytometry. Exosomes derived from all three cell lines were found to be positive (relative fluorescence intensity (RFI) > 1), but the CD16 RFI values differed between the single cell lines (Figure 2B). While the THP-1 derived exosomes carried high levels of CD16 (RFI = 3.7 ± 1.6), PCI-30 and SCC-47 derived exosomes carried consistently low levels of CD16 (RFI = 1.7 ± 0.6 and 1.6 ± 0.6). Overall, the presence of CD16 on exosomes derived from both monocytic and tumor cells provided the basis for investigating levels of CD16 on exosomes from HNSCC patients to examine their role in modulating the TME.

### 2.3. Clinicopathological Characteristics of HNSCC Patients

The clinicopathological characteristics of the HNSCC patients (n = 53) whose plasma was used for exosome isolation are listed in Table 1. The mean age was 62 years with a range between 36 and 84 years. The majority of patients (81%) were male. The primary tumor was located in the oral cavity (43%), pharynx (28%), or larynx (28%). As determined by clinical evaluations, 42 patients (79%) were considered having an active disease (AD), whereas 11 patients (21%) were considered having no evidence of disease (NED). Half of the patients (49%) presented with advanced tumor stage T3/4 and 68% had a lymph node metastasis. Forty-three percent of the patients were Union for International Cancer Control (UICC) stage I or II and 57% were UICC stage III or IV. The human papillomavirus (HPV) status, routinely determined by p16 immunohistochemistry, was positive in 10 patients, negative in 17 patients, and not evaluated in 26 patients. At the time of diagnosis, 70% respectively 87% of the patients consumed alcohol or tobacco. HDs (n = 7) were matched for gender and age.

### 2.4. Exosomes from HNSCC Carry Surface CD16

**Figure 3:**
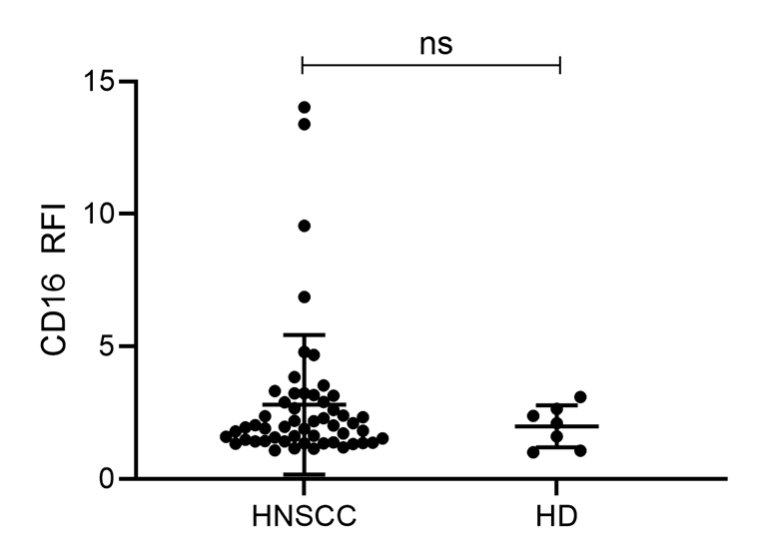
CD16 levels on plasma-derived exosomes from HNSCC patients and HDs. Total exosomes from HNSCC patients (n = 53) or HDs (n = 7) were stained for CD16 and surface levels as determined by on-bead flow cytometry are shown as RFI compared to an appropriate isotype control. Bars represent mean with SD. *p* values were determined by Mann–Whitney test, with ns = not significant.

First, CD16 surface levels of total exosomes were compared between HNSCC patients and HDs. HNSCC patients had slightly, but not significantly, elevated CD16 levels compared to HDs (Figure 3). Although all HNSCC patients had CD16 positive exosomes, some patients had highly elevated levels. To our knowledge, this is the first study to show that exosomes isolated from plasma carry surface CD16.

**Figure 4:**
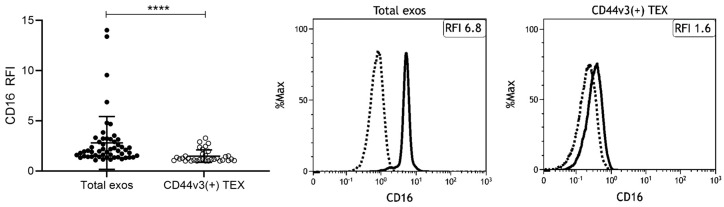
CD16 levels on plasma-derived exosomes from HNSCC patients. Total exosomes or CD44v3(+) tumor-derived exosomes (TEX) isolated from plasma of HNSCC patients (n = 53 or n = 33) were stained for CD16. Surface levels as determined by on-bead flow cytometry are shown as RFI compared to an appropriate isotype control. Bars represent mean with SD. *p* values were determined by Mann–Whitney test, with **** corresponding to *p* ≤ 0.0001. Representative flow cytometry histograms depicting CD16 levels on total exosomes and CD44v3(+) TEX are shown. The solid line represents the CD16 signal, the dashed line represents the isotype control.

As CD44v3 allows for enrichment of TEX, which are highly present in plasma of HNSCC patients, immune capture by CD44v3 was performed prior to CD16 surface staining. Interestingly, total exosomes from HNSCC patients had significantly higher CD16 levels compared to CD44v3(+) TEX (Figure 4). This might indicate that CD16 is rather present on exosomes derived from other cell populations such as immune cells. These results are in line with the findings in cell-line derived exosomes: Exosomes derived from tumor cell lines showed lower CD16 levels compared to exosomes derived from monocytic cells (Figure 2).

### 2.5. Correlation of CD16 Surface Levels on Exosomes with Clinicopathological Parameters

**Figure 5:**
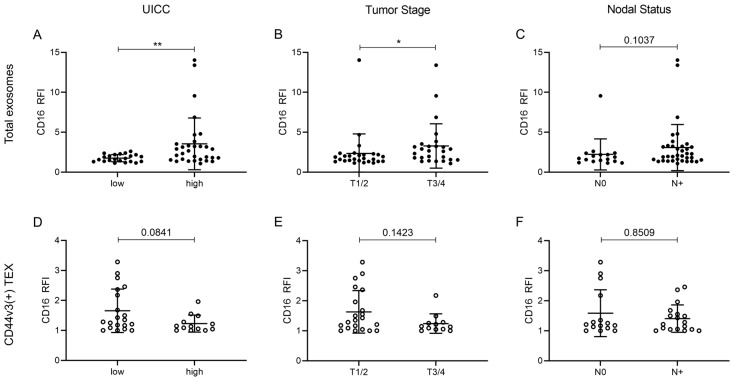
Correlations of CD16 levels on plasma-derived total exosomes and CD44v3(+) TEX with clinicopathological data. Significant correlations were observed between CD16 levels on total exosomes and UICC status (**A**) as well as tumor stage (**B**). No significant correlation was observed between CD16 levels on total exosomes and nodal status (**C**). No significant correlations were observed between CD16 levels on CD44v3(+) TEX and UICC status (**D**), tumor stage (**E**), or nodal status (**F**). Results are shown as RFI. Bars represent mean with SD from n = 53 (**A**–**C**) and n = 33 (**D**–**F**) HNSCC patients. *p* values were determined by Mann–Whitney test, with * and ** corresponding to *p* ≤ 0.05 and *p* ≤ 0.01, respectively.

The CD16 surface levels on total and TEX enriched CD44v3(+) exosomes were examined for correlation with clinicopathological data. Therefore, patients were stratified according to their UICC grade (low [I/II] vs. high [III/IV]), tumor stage (T1/2 vs. T3/4), and nodal status (N0 vs. N > 1). CD16 surface levels were significantly higher on total exosomes of UICC high stage patients compared to UICC low stage patients (Figure 5A). They were also significantly increased on total exosomes of patients with advanced tumor stages T3/4 compared to T1/2 (Figure 5B). Patients with evidence of nodal metastasis exhibited elevated CD16 surface levels on total exosomes, albeit not significant (Figure 5C). No significant differences were seen when the data were correlated with disease status, HPV status, and primary tumor site. CD16 surface levels on CD44v3(+) TEX showed no significant clinicopathological correlations, although a trend was visible towards patients with a less aggressive tumor profile (Figure 5D–F): Patients with low UICC stage exhibited elevated CD16 surface levels on CD44v3(+) TEX, an inverse picture compared to the levels on total exosomes (Figure 5A,D).

## 3. Discussion

Emerging data suggest that plasma of HNSCC patients is rich in exosomes derived from both tumor cells and immune cells [16,19]. While TEX might serve as biomarkers for tumor status, exosomes produced from immune cells can serve as biomarkers for immune dysfunction [30,31]. To explore exosome-mediated immune modulation within the TME, we asked whether the Fcγ receptor CD16 is present on exosomes from plasma of HNSCC patients, whether CD16 is differentially present on TEX compared to total exosomes, and if CD16 levels on exosomes correlate with clinicopathological parameters.

This study showed that plasma-derived exosomes from tumor patients carry CD16 on their surface. Additionally, levels of CD16 were similar on exosomes from HNSCC patients and HDs, although a trend was visible towards higher CD16 levels on exosomes from HNSCC patients. As the exosome population from plasma represents a mixture of exosomes derived from different cell types, we enriched TEX by immune capture using CD44v3, a protein commonly overexpressed and associated with poor outcome in HNSCC [22,23]. It was visible that TEX do not remarkably account for the high levels of CD16 on total exosomes which we observed in HNSCC patients. Although TEX exhibited certain levels of surface CD16, total exosomes displayed much higher levels. Hence, we suppose that CD16 is found on exosomes derived from CD16 expressing immune cells, such as monocytes, neutrophils, NK cells, or T cells [7]. This is supported by our finding that the monocytic THP-1 cell line and its corresponding exosomes had higher CD16 levels compared to HNSCC cell lines. Based on the current data, we are not able to distinguish from which immune cell population the CD16 positive exosomes arise and to which extent they present a mixture derived from different cell populations. However, results from our and other laboratories [32] emphasize the expression of CD16 on a subset of monocytes. Both non-classical and intermediate monocytes are characterized by CD16 expression [33,34] and accordingly represent a source for CD16 positive exosomes. Other studies report the ability of T cell subsets to express CD16 and as such to contribute to ADCC [35,36,37]. This is intriguing as our group showed previously that T cell-derived exosomes comprise around 40%–50% of total exosomes isolated from plasma of HNSCC [19]. Thus, T cell-derived exosomes depict a great pool for CD16 positive exosomes. Another prominent immune cell subset expressing CD16 are NK cells which are the main mediators of ADCC in physiological and therapeutic settings [38]. CD16 is indispensable for functional ADCC, as downregulation or blocking of CD16 on NK cells has been shown to decrease NK cell activity thereby promoting tumor survival [39,40]. In HNSCC, altered NK cell functions strongly contribute to the immune suppressive, pro-tumorigenic TME but the underlying mechanisms are not yet fully understood. Studies suggested reduced NK cell numbers expressing CD16 [2,41], downregulated CD16 on NK cells [40,42], or an imbalance of different NK cell subpopulations [4,5,43]. However, the importance of CD16 for a functional ADCC is undisputed.

The presented results of increased CD16 levels on plasma-derived exosomes from HNSCC patients suggest that CD16 positive exosomes might emerge as mediators of immune suppression. As such, CD16 positive exosomes—independent from which immune cell population they derive—might mimic NK cells in their function of cross-linking with antibody-coated malignant cells without exerting their cytotoxic function. Thereby, malignant cells are able to escape from NK cell-mediated ADCC. What appears as a discrepancy, may result in the same effect: While CD16 expression on NK-cells is anti-tumorigenic and thus reduced in HNSCC [40,42], high levels of CD16 on exosomes—as particularly observed in patients with advanced disease—seem to be pro-tumorigenic as competition of CD16^high^ exosomes with CD16^low^ NK cells for antibody coated malignant cells might result in massive, synergistic immune suppression.

The therapeutic mAB cetuximab, an epidermal growth factor receptor inhibitor, is approved for the treatment of locoregionally advanced and recurrent/metastatic HNSCC and colon carcinoma. One of its effects is the induction of NK cell function by mediating ADCC in a CD16 dependent manner and thereby boosting the anti-tumor immune response in cancer patients [44,45,46]. Even more, the importance of CD16 in reducing cetuximab efficiency in esophageal squamous cell carcinoma patients has been emphasized before [40]. Thus, the presence of CD16 on circulating exosomes might reduce the efficacy of therapeutic mABs by trapping them via their Fc part and preventing a proper accumulation in the TME. This effect of antibody-capturing by circulating exosomes has been described before in the context of immune checkpoint inhibition with trastuzumab [47,48] and anti-PD-L1 antibodies [49,50].

We found that CD16 levels on total exosomes (representing all cell populations of the TME), but not on TEX, significantly correlated with tumor stage and tumor aggressiveness. Thus, CD16 on total exosomes might serve as an indicator of the grade of immune suppression in HNSCC. While the immune suppression mediated by CD16 positive exosomes is not very pronounced in early disease, it is manifested in advanced and aggressive disease possibly due to reduced ADCC. This is in line with previous reports showing that the level of immune suppression differs between tumor stages and that advanced HNSCCs are generally more immune suppressive than early-staged malignancies [51,52,53]. The relevance of immune cell-derived exosomes in the TME has been highlighted in our previous studies demonstrating not only an immune suppressive cargo but also a significant correlation with response to therapy [18,19].

Overall, we demonstrated that exosomes have the potential to serve as easily accessible, non-invasive biomarkers for tumor status and tumor aggressiveness as well as for the degree of immune suppression in HNSCC patients. As such, CD16 positive exosomes might contribute to immune suppression by preventing ADCC.

## 4. Materials and Methods

### 4.1. Cell Lines

The HNSCC cell lines PCI-30 and UM-SCC-47 (SCC-47, a gift from Dr. Theresa L Whiteside, University of Pittsburgh, PA, USA) were established, characterized, and maintained as described before [54] and were cultured in DMEM (Gibco, Carlsbad, CA, USA, 41966-029) supplemented with 10% exosome-depleted fetal bovine serum (FBS, Gibco, Carlsbad, CA, USA, A2720801) and 1% penicillin-streptomycin. The monocytic THP-1 cell line was purchased from the American Type Culture Collection (ATCC) and was maintained in RPMI (Gibco, Carlsbad, CA, USA, 21875-034) supplemented as described above.

### 4.2. Patients

Peripheral blood specimens were randomly obtained from 53 HNSCC patients and 7 HDs after informed consent was obtained from each individual. The collection of blood samples was approved by the Ethics Committee of the University of Ulm, Helmholtzstraße 20, 89081 Ulm (#90/15). The HNSCC patients were seen at the Department of Otorhinolaryngology, Head and Neck Surgery of the University of Ulm from 2015 till 2018. Blood samples were centrifuged at 1000× *g* for 10 min. Plasma specimens were stored in aliquots at −80 °C. Table 1 provides the clinicopathological characteristics of the patients included in this study.

### 4.3. Exosome Isolation by Mini Size Exclusion Chromatography (Mini-SEC)

For exosome isolation, mini size exclusion chromatography (mini-SEC) was used as previously described [28]. Briefly, freshly thawed plasma samples or cell culture supernatants were sequentially centrifuged at 2000× *g* for 10 min at room temperature (RT) and 10,000× *g* for 30 min at 4 °C, followed by filtration through 0.22 μm syringe-filters (Millipore, Burlington, MA, USA, SLGPO33RB). Cell culture supernatants were then concentrated to 1 mL using Vivacell 100 filter units (Sartorius, Göttingen, Germany, VC1042). Aliquots (1 mL) of plasma or concentrated cell culture supernatants were loaded on mini-SEC columns and eluted with PBS. Sequential 1 mL fraction #4 was collected and used for further analysis as it was described to be highly enriched in exosomes [28].

### 4.4. BCA and Exosome Concentration

To determine the protein concentration of the isolated exosome fraction, Pierce BCA Protein Assay Kit (ThermoFisher Scientific, Waltham, MA, USA, 23225) was applied according to manufacturer’s protocol. Exosomes were concentrated using 100 kDa cutoff centrifugal filter units (Millipore, Burlington, MA, USA, UFC5100BK). For on-bead flow-cytometry, 10 µg of exosomes in 100 µL PBS were used, for Western blot analysis, 20 µg of exosomes in 40 µL PBS were used.

### 4.5. Characterization of Exosomes

Freshly isolated exosomes were prepared for TEM by negative staining. Therefore, 5 µL of the exosome solution was added onto a glow discharged carbon-coated copper TEM grid and incubated for 1 min. Then, the grids were washed 3 times with a droplet of water. Next, the grids were incubated three times with droplets of 3% uranyl acetate in water for 30 s each. Afterward, the grids were dried on air with a small amount of uranyl acetate left. The grids were imaged with a JEOL 1400 TEM (Freising, Germany).

NTA was performed using ZetaView PMX-220 (Particle Metrix, Inning am Ammersee, Germany). Freshly isolated exosomes were diluted 1:100,000 in 1 mL PBS and loaded into the cell. Measurements were performed at 11 different positions throughout the cell, with three cycles of records at each position. Instrument parameters were set to a temperature of 25 °C, a sensitivity of 85 and a shutter of 100. PBS and 100 nm polystyrene beads were used as controls. For data recording and for calculating nanoparticle size ranges and concentrations, corresponding ZetaView 8.05.11 software was used.

For Western blots, 20 µg exosomes/lane were mixed at a *v*/*v* ratio of 1:5 with Pierce Lane Marker Reducing Sample Buffer (ThermoFisher Scientific, Waltham, MA, USA, 39000) for detection of CD9 and TSG101 or with Pierce Lane Marker Non-Reducing Sample Buffer (ThermoFisher Scientific, Waltham, MA, USA, 39001) for detection of CD63 and CD81 and denatured for 5 min at 95 °C. Samples were separated on 12% Mini-Protean TGX Precast Gels (BioRad, Hercules, CA, USA, 4561044) and transferred to nitrocellulose membranes using the Trans-Blot Turbo Transfer System (BioRad, Hercules, CA, USA). Membranes were incubated overnight at 4 °C with the following primary antibodies: anti-TSG101 (Invitrogen, Carlsbad, CA, USA, PA5-31260, 1:500), anti-CD9 (Invitrogen, Carlsbad, CA, USA, 10626D, 1:1000), anti-CD63 (Invitrogen, Carlsbad, CA, USA, 10628D, 1:1000) and anti-CD81 (Invitrogen, Carlsbad, CA, USA, 10630D, 1:1000). Horseradish peroxidase (HRP)-conjugated secondary antibodies (ThermoFisher Scientific, Waltham, MA, USA, 31450 and 31460, 1:10,000) were added for 40 min at RT. Bands were detected using SuperSignal West Dura Extended Duration Substrate (ThermoFisher Scientific, Waltham, MA, USA, 34076) and ChemiDoc XRS+ Imaging System (BioRad, Hercules, CA, USA).

These methods for exosome characterization are in line with the MISEV 2018 guidelines for the definition of extracellular vesicles [29] and are routinely performed as described in detail in our previous publications [16,26,27].

### 4.6. Flow Cytometry of Cells

Cultured cells were detached from flasks using TrypLE Express Enzyme (Gibco, Carlsbad, CA, USA, 12605-010) and washed twice with FACS buffer (PBS supplemented with 2% bovine serum albumin (BSA)). 10^6^ cells were stained in 100 µL FACS buffer using 5 µL of PE-Cy7 conjugated anti-CD16 (BD, Franklin Lakes, NJ, USA, 335788) for 30 min at 4 °C in the dark. After two further washing steps, cells were measured in a Gallios flow cytometer using Kaluza 1.0 (Beckman Coulter, Brea, CA, USA) and data were analyzed using Kaluza Analysis 2.1 software.

### 4.7. Immune Capture and On-Bead Flow Cytometry of Exosomes

Exosomes were first captured on ExoCap Streptavidin magnetic beads (MBL Life Science, Woburn, MA, USA, MEX-SA) as previously described [18,19,21]. Briefly, exosomes (10 µg in 100 µL PBS) were incubated for 2 h at RT with biotin-labeled anti-CD63 (BioLegend, San Diego, CA, USA, 353018) for total exosome capture or with biotin-labeled anti-CD44v3 (R&D Systems, Minneapolis, MN, USA, BBA11) for the enrichment of TEX. Biotin-labeled anti-CD63 was adjusted to a concentration of 1 µg in 100 µL PBS. Anti-CD44v3 was custom biotinylated using Lightning-Link Rapid Biotin Antibody Labeling Kit (Novus Biologicals, Littleton, CO, USA, 370-0010) and used at 2 µg in 100 µL PBS. Next, an aliquot of beads (10 µL for CD63, 100 µL for CD44v3) was added and samples were again incubated for 2 h at RT. The uncaptured fraction was removed and samples were washed using a magnetic rack.

For CD16 detection by on-bead flow cytometry, the bead/anti-CD63 or anti-CD44v3/exosome complexes were incubated with 5 µL of PE-Cy7 conjugated anti-CD16 for 1 h at RT. An appropriate isotype control (BD, Franklin Lakes, NJ, USA, 348788) was used. Next, the stained complexes were washed three times with PBS using a magnetic rack and were finally resuspended in 300 µl PBS for flow cytometry. Detection was performed using a Gallios flow cytometer with Kaluza 1.0 software (Beckman Coulter, Brea, CA, USA). Samples were run for 2 min and around 10,000 events were acquired. Gates were set in the bead fraction visible in the forward/sideward light scatter. Data are presented as RFI which equals the mean fluorescence intensity (MFI) of the stained sample divided by the MFI of the isotype control.

## 5. Conclusions

We report for the first time that CD16 surface levels on total exosomes, but not on CD44v3(+) TEX, correlate with clinicopathological variables in HNSCC patients and could therefore be considered as liquid biomarkers for tumor status and aggressiveness.

## Figures and Tables

**Table 1:** Clinicopathological parameters.

Characteristics	Patients (n = 53)	
	n	%
**Age (years)**		
≤60	24	45
>60	29	55
(range: 36–84)		
**Gender**		
Male	43	81
Female	10	19
**Disease status**		
AD	42	79
NED	11	21
**Primary tumor site**		
Oral cavity	23	43
Pharynx	15	28
Larynx	15	28
**Tumor stage**		
T1	13	25
T2	14	26
T3	8	15
T4	18	34
**Nodal status**		
N0	17	32
N1	16	30
N2	14	26
N3	6	11
**Distant metastasis**		
M0	53	100
**UICC stage**		
I	16	30
II	7	13
III	10	19
IV	20	38
**HPV status**		
Positive	10	19
Negative	17	32
Undefined	26	49
**Alcohol consumption**		
Yes	37	70
No	14	26
Unknown	2	4
**Tobacco consumption**		
Yes	46	87
No	7	13

AD: active disease, NED: no evidence of disease, UICC: Union for International Cancer Control, HPV: Human papillomavirus.

## Abbreviations

AD	Active disease
ADCC	Antibody-dependent cell-mediated cytotoxicity
ApoA1	Apolipoprotein A1
EV	Extracellular vesicle
FcR	Fc receptor
Grp94	Glucose-regulated protein 94
HD	Healthy donor
HNSCC	Head and neck squamous cell carcinoma
HPV	Human papilloma virus
mAB	Monoclonal antibody
MFI	Mean fluorescence intensity
NED	No evidence of disease
NK	Natural killer
NTA	Nanoparticle tracking analysis
RFI	Relative fluorescence intensity
RT	Room temperature
SEC	Size exclusion chromatography
TEM	Transmission electron microscopy
TEX	Tumor-derived exosomes
TME	Tumor microenvironment
TSG101	Tumor susceptibility gene 101
UICC	Union for International Cancer Control
